# Effects of Addition of Mannitol Crystals on the Porosity and Dissolution Rates of a Calcium Phosphate Cement

**DOI:** 10.6028/jres.115.016

**Published:** 2010-08-01

**Authors:** Debra Vazquez, Shozo Takagi, Stan Frukhtbeyn, Laurence C. Chow

**Affiliations:** National Institute of Standards and Technology, Gaithersburg, MD 20899-0001

**Keywords:** calcium phosphate cement, dissolution rate, mannitol crystal, mechanical properties, porosity

## Abstract

The bone defect repair functions of calcium phosphate cement (CPC) are related to its osteoconductivity and its gradual replacement by new bone. Adding mannitol to CPC may enhance its bone repair potential by increasing CPCs macroporosity and dissolution rate. The objective of the study was to assess microporosity and macroporosity and dissolution rates for CPC mixed with mannitol. Three groups of CPC discs were prepared by combining an equimolar mixture of tetracalcium phosphate and anhydrous dicalcium phosphate with (0 %, 10 %, or 50 %) mass fraction (hereafter expressed as mass %) of mannitol. Macroporosity and microporosity of the samples were calculated from volume and mass measurements of the discs. Discs were then placed in a pH 3.0 demineralizing solution simulating acidified physiological solution, and dissolution rates were measured by a previously described constant-composition titration method. Pure CPC exhibited no macropores and microporosity (mean ± s.d.; n = 5) of (46.8 ± 0.8) % volume fraction (hereafter expressed as vol %). Adding 10 mass % mannitol resulted in 15.6 ± 3.9 vol % macroporosity and 39.4 ± 1.8 vol % microporosity, and adding 50 mass % mannitol produced 54.7 ± 0.8 vol % macroporosity and 21.1 ± 0.4 vol % microporosity. The dissolution rates (mean ± s.d.; n = 5) of CPC with (0, 10, and 50) mass % mannitol incorporation were (30.6 ± 3.4, 44.8 ± 10.2, and 54.7 ± 3.6, respectively) μg · cm^−2^ · min^−1^, or (0.018 ± 0.002, 0.032 ± 0.007, and 0.072 ± 0.005, respectively) μL · cm^−2^ · min^−1^. Adding either 10 mass % or 50 mass % mannitol into CPC significantly (p < 0.05) increased CPC dissolution rates. Adding mannitol readily increased macroporosity and dissolution rate of CPC, which may enhance the capacity of CPC to be osteoconductive.

## 1. Introduction

Calcium phosphate cements (CPC) have become a subject of much interest in dental and medical materials research because of their excellent biocompatibility. These cements are self-hardening and form hydroxyapatite (HA) or carbonated HA as the end product [1–4]. A calcium phosphate cement that initially contains an equimolar mixture of tetracalcium phosphate (TTCP), Ca_4_(PO_4_)_2_O, and dicalcium phosphate anhydrous (DCPA), CaHPO_4_, hardens about 30 min after mixing the powder with water [4]. Animal study results showed that CPC resorbed slowly and was replaced by new bone [5–7]. However, for certain clinical applications a more rapid resorption and replacement by new bone is desirable. Previous studies have shown that ceramic calcium phosphate implants with macropores (> 100 μm in diameter) allowed ingrowth of bone tissue with functional haversian systems and facilitated osseointegration [8–11].

The formation of macropores in CPC can be achieved by incorporation of water-soluble porogens such as sucrose, NaHCO_3_ and NaH_2_PO_4_ crystals into CPC [12]. Mannitol, CH_2_OH(CH(OH))_4_CH_2_OH, crystals are also a good candidate as a source of the water-soluble porogen [13–16]. Although a previous animal study showed that a macroporous TTCP+DCPA-based CPC was resorbed and replaced by bone more rapidly than the same CPC implant without macrospores, no studies have been carried out to compare the dissolution properties of this CPC with and without macrospores in a simulated resorption environment [17].

Direct measurements of the extra cellular fluid from bone-resorbing cells showed the pH to be between 5.0 and 3.0 [18]. The *in vitro* dissolution rates of different calcium phosphates and set CPC products were studied using a dual constant composition titration method to predict *in vivo* resorption properties [19]. In this study, standard dicalcium phosphate dihydrate (DCPD), CaHPO_4_ · 2H_2_O, dissolved three times faster than HA (p < 0.05). CPC dissolved 1.2 times faster than HA but the difference was not statistically significant (p > 0.05). This method can be used to study the rate and stoichiometry of dissolution of calcium-phosphate bone-graft materials and coatings under a wide range of mineral saturation conditions [19].

The purpose of this study was to determine the effects of macroporosity on dissolution rate of CPC.

## 2. Materials and Methods

### 2.1 Materials and Sample Preparation

TTCP was prepared by heating an equimolar mixture of commercially obtained DCPA and CaCO_3_ (J. T. Baker Chemical Co., NJ, USA)^1^ at 1500 °C for 6 h in a furnace (Model 51333, Lindberg, Watertown, WI) and quenched at room temperature. The TTCP was crushed and ground in a ball mill (Retsch PM4, Brinkman) for 24 h in cyclohxane. DCPA was ground for 24 h in the ball mill in ethanol and then dried in air. The CPC powder used in the study consisted of an equimolar mixture of TTCP and DCPA with median particle sizes of 5 μm and 1 μm, respectively. The particle sizes of TTCP and DCPA were measured using a centrifugal particle size analyzer (SA-CP3, Shimadzu, Kyoto, Japan) with an estimated standard uncertainty of 0.2 μm. Mannitol, CH_2_OH(CHOH)_4_CH_2_OH, (Sigma) was recrystallized in ethanol/water, filtered, dried, ground, and collected between sieves with openings of 250 μm (top sieve) and 125 μm (bottom sieve). The collected mannitol crystals typically contain prismatic and rod-shaped crystals with lengths ranging from ≈100 μm to ≈400 μm.

Three CPC mixtures containing mass fractions of (a) 0 %, (b) 10 %, and (c) 50 % mannitol were prepared. A 0.5 mol/L Na_2_HPO_4_ solution saturated with respect to Na_2_HPO_4_ was used as the cement liquid. The method used for preparing specimens was described previously [19]. Because the ground TTCP was used for the mixtures, low powder-to-liquid (P/L) ratio was used for obtaining a good consistency, such as P/L = 2.5, where the powder refers to the amount of CPC powders in CPC-mannitol mixture, was used for preparing specimens in all cases. After mixing, the cement paste was packed into a stainless steel mold [20] kept for 4 h in 100 % relative humidity at 37 °C. The sample was then, removed from the mold and placed in distilled water at 37 °C for 20 h during which the mannitol was dissolved, forming pores in the shape of the crystals. The set specimen had a dimension of 6 mm diameter and approximately 3 mm height.

For density measurements, samples were dried at 70 °C for 4 h and weighed. For dissolution rate measurements, each sample disc was mounted on a plexiglas^®^ rod with sticky wax (Moyco Industries, Philadelphia, PA), leaving one flat surface and the perimeter areas exposed to the solution (total exposed area). The mounted specimen was soaked in 5 mL of the demineralizing solution for 1 h prior to the dissolution experiment to allow the specimen to be fully hydrated. The porosity was calculated from the bulk density of the sample using a previously established procedure [12].

### 2.2 Measurement of Density and Porosity

The inherent microporosities (I-MicP) on the pure cement samples (prepared without the porogen) due to the volume taken up by the cement liquid can be calculated by the following equation:

(1) $$\text{I-MicP}=((d_{\text{HA}}-d_{\text{cpc}})/d_{\text{HA}})\times 100\%.$$

Where d_HA_ = 3.14 g/cm^3^ is the crystal density of HA [21] and d_cpc_ is the density of the dried additive-free sample, which is calculated by the equation:

(2) $$d_{\text{cpc}}=m_{\text{cpc}}/V\text{.}$$

Where m_cpc_ is the mass, V = πr^2^h is the volume, and r is the radius and h is the height of the sample.

Samples prepared with mannitol can be considered to consist of a pure CPC phase and macropores (MacP) formed by dissolution of the additive crystals. The MicP of the CPC phase in the sample should essentially be the same as the I-MicP of the additive-free samples. The MacP of the sample, expressed as volume fraction in percent (%), can be calculated by the equation:

(3) $$\text{MacP}=(1-d_{\text{cpc-add}}/\text{mean}\;d_{\text{cpc}})\times 100\%.$$

Where d_cpc-add_ = m_cpc-add_/V is the density and m_cpc-add_ is the mass of the water-extracted, dried sample prepared with an additive. Mean d_cpc_, the average density of the additive-free CPC samples, is the average of the d_cpc_ values calculated from Eq. (2) for samples in group (a) that contained 0 % mannitol.

The microporosity, MicP, of a mannitol-containing sample can be calculated by equation:

(4) $$\text{MicP}=(d_{\text{cpc-add}}/\text{mean}\;d_{\text{cpc}})\times \text{mean}\;\text{I-MicP}.$$

Where mean I-MicP is the averaged I-MicP value calculated by Eq. (1) of all the samples in group (a). Combining Eqs. 3 and 4 lead to a direct relationship between MicP and MacP as expressed by the equation:

(5) MicP=((100−MacP)×meanI-MicP)/100.

### 2.3 Dissolution Rate Measurements

The dissolution rate data was measured using a simplified *in vitro* dual constant-composition titration system [19].

#### 2.3.1 Demineralizing Medium

The lowest pH reported for the extra cellular fluid of osteoclast cells was about 3.0 while the pH of the fluid in direct contact with the reabsorbing bone was considerably higher, e.g., about 5 [22]. Thus, demineralizing solutions with pH’s in the range of 3.0 to 5.0 should be relevant to the *in vivo* resorption process. In this study, a pH 3.0 demineralizing solution was used so that the dissolution process would proceed more rapidly and the dissolution experiment could be conducted more expeditiously. The demineralizing solution had a composition ([Ca(OH)_2_] = 1.15 mmol/L; [H_3_PO_4_] = 1.2 mmol/L; [KCl] = 133 mmol/L) that is similar to the ionic composition of serum [6] except for the pH, which was adjusted to 3.0 by adding HCl(2.69 mmol/L). The solution was designed to have little buffer capacity so that a small amount of CPC dissolution would raise the pH, which, in turn, would trigger the titration process as described below.

#### 2.3.2 Titrant Solution

The composition of the titrant solution was the same as the demineralizing medium except that the amounts of Ca(OH)_2_ and H_3_PO_4_, equivalent to 0.23 mmol/L of CPC (using hydroxyapatite (HA), Ca_5_(PO_4_)^2^ (OH), as the formula) were subtracted. Thus, the titrant composition was 0 mmol/L Ca(OH)_2_, 0.51 mmol/L H_3_PO_4_, 2.69 mmol/L HCl, and 133 mmol/L of KCl, and had a pH of 2.69.

#### 2.3.3 Principle of Operation

Results from the previous dual-constant composition study [18] showed that dissolution of a given CPC sample occurred at an essentially constant Ca/P ratio that corresponded to the bulk Ca/P ratio of the sample. Thus, the dissolution rate can be reliably measured using a single constant composition method, which had been used previously for measuring the rate of enamel demineralization [23]. When applied to dissolution of calcium phosphate materials, this technique is based on the principle that dissolution of most calcium phosphates, such as HA, consumes protons (H^+^ ion). As a result, dissolution of a small amount of a CPC sample in the demineralizing solution, which as described above has little buffer capacity, will cause an immediate increase in pH. In response, the titrator will add an appropriate amount of the titrant solution to keep the pH at the set point (pH 3.0 in this case). Since the titrant is the equivalent of the demineralizing medium with 0.23 mmol/L CPC subtracted, it can be shown that titrant addition will maintain the demineralizing medium composition constant when the pH is kept constant at 3.0.

#### 2.3.4 Experimental Procedure

**Fig. 1:**
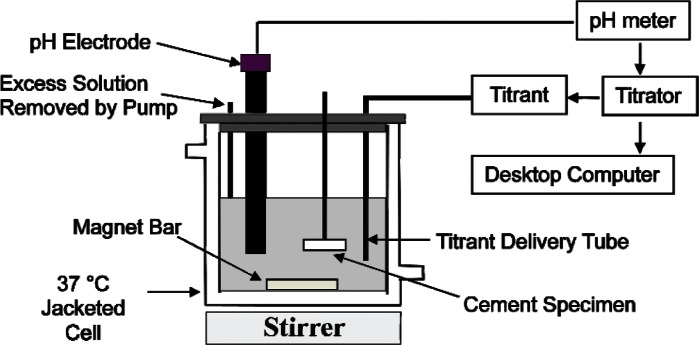
Schematic drawing of the single constant composition system.

The schematic drawing of the titration system for the dissolution rate measurements is shown in Fig. 1. Each titration experiment was conducted in a jacketed, 100 mL capacity glass vessel connected to a circulating bath set at 37 °C. A pencil-size combination pH electrode was used as the sensor for triggering the delivery of the titrant solution by an automatic titrator (Dosimat 665, Brinkman Instruments, Westbury, NY). Throughout the experiment, a desktop PC equipped with an analog-to-digital converter card (Model BNC-2080, National Instruments, Austin, TX) recorded one reading per sec of the mV signals (corresponding to the pH) from the electrode and the mV signals (corresponding to the volume of titrant added) from the titrator. The demineralizing solution (40 mL) was placed in the vessel, and a stable pH electrode reading was obtained under constant stirring (400 rpm). This reading was used as the set point on the titrator. Addition of the titrant would be triggered when the pH electrode mV reading was more negative than the set value, indicating that the pH was greater than the initial set value of 3. A CPC sample, attached to a plexiglas^®^ rod, was then placed in the demineralizing solution. A small amount of dissolution of the sample causes an increase in the pH, and triggers the addition of the titrant, which is more acidic than the demineralizing solution, to keep the pH at 3.0. At the same time, titrant addition dilutes the demineralizing solution with respect to both the [Ca] and [P] to keep the composition of the demineralizing solution also constant. A small suction tube connected to a peristaltic pump (Masterflex, Cole-Palmer Instrument Company, Chicago, IL) was placed in the vessel to remove liquid above a preset level of 60 mL so that the volume of the solution was also kept constant throughout the rest of the dissolution process. Dissolution rate measurements were conducted on three samples from each of the three experimental groups.

#### 2.3.5 Interpretation of Titration Results

The cumulative amount of dissolution (in the unit of mmol/cm^2^) expressed as the amount of calcium, M_Ca_(*t*) dissolved per square centimeter of sample surface area from the beginning of the experiment to time *t* (in minutes), was calculated from the volume (in the unit of L) of the titrant, V_ca_(*t*), added during the time interval using the equation:

(6) $$M_{\text{Ca}}(t)=V_{\text{Ca}}(t)\times [\text{Ca}]/A$$

where [Ca] = 1.15 mmol/L is the difference between the calcium concentrations in the demineralizing solution and the titrant, and A = 0.283 cm^2^ is the sample surface area exposed to the demineralizing solution. The average dissolution rate (in the unit of nmol/min cm^2^) for the same period, expressed as the amounts of calcium dissolved per cm^2^ of sample surface area per min, R_Ca_(*t*), is calculated from the equations:

(7) $$R_{\text{Ca}}(t)=M_{\text{Ca}}(t)\times 10^{6}/t.$$

The average dissolution rate (in the unit of μg/min-cm^2^), R_M_ (*t*), may be expressed as the mass of HA, dissolved per cm^2^ of sample surface area per min using the equation:

(8) $$R_{M}(t)=(R_{\text{Ca}}(t)\times \text{FW})/(N\times 10^{3})$$

where FW = 502 is the formula weight and N = 5 is number of Ca atoms in the formula of HA.

The rate of implant volume loss (μL/cm^2^ × min) was calculated using the following equation:

(9) $$((R_{m}(t)\times 10^{-6})/d_{\text{specimen}})\times 1000.$$

Where d_specimen_ was the density of specimen (d_cpc_ or d_cpc-add_).

#### 2.3.6 Statistical Analysis

A commercially obtained statistical analysis software, Kwikstat, (Texas Soft, Cedar Hill, TX), was used to perform ANOVA on porosities (MicP and MacP values) and dissolution rate (R_m_) data to determine significant differences among the groups. In the cases where a significant difference was detected, Newman-Keuls multiple comparison tests were conducted. In this study, the standard deviation is considered as the standard uncertainty for all experimentally measured values.

## 3. Results

The mean density, MacPs, MicPs and total porosities (mean ± sd; n = 5) are shown in Table 1. All values within a column are significantly different (p < 0.05). It can be seen that because a relatively low powder/liquid (P/L) of 2.5 was used in CPC specimen preparation, the mannitol-free material had a high inherent microporosity (MicP) of 46.8 % compared to about 34 % when a P/L of 4 was used [24]. Inclusion of 10 % mass fraction (hereafter expressed as mass %) and 50 mass% mannitol created macroporosity (MacP) of 15.6 % volume fraction (hereafter expressed as vol %) and 54.7 vol %, respectively. Because these materials had smaller amounts of pure CPC phase per unit volume than the pure CPC sample (0 mass % mannitol), the former had lower MicP values of 39.4 vol % and 21.1 vol %, respectively compared to 46.8 vol %. The dissolution rates expressed as mass loss and volume loss (mean ± sd; n = 5) are shown in Table 2. All values within a column are significantly different (p < 0.05).

The results showed that an increase in MacP significantly increased the dissolution rate, especially in terms of the rate of volume loss.

**Fig. 2:**
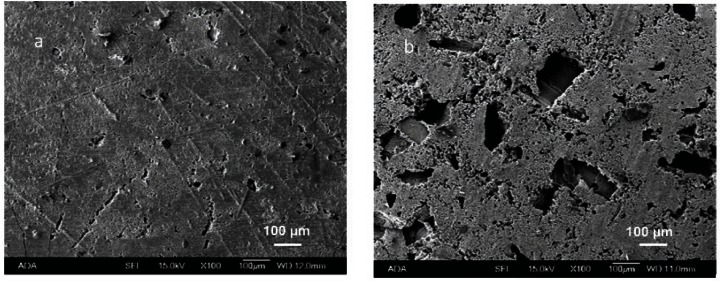
Surface morphology of CPC specimens containing 0 mass % and 50 mass % mannitol crystals (a and b, respectively).

The SEM picture shows the shapes of mannitol crystals in the 50 mass% mannitol containing CPC (Fig. 2)

## 4. Discussion

In the present study the cement liquid was a 0.5 mol/L Na_2_HPO_4_ solution pre-saturated with mannitol crystals such that little or no mannitol crystal dissolution would occur as the cement powder and liquid were mixed. This helped produce macropores in the same shapes and sizes of the mannitol crystals originally present in the cement powders (Fig. 2). This method should be generally useful for producing pores in CPC materials by incorporating soluble porogens.

**Fig. 3:**
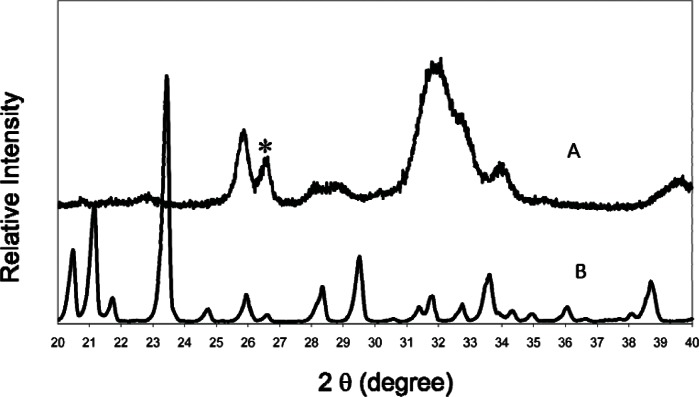
Powder x-ray diffraction patterns of CPC specimen with 50 mass % mannitol after immersion in water for 1 d (A), and mannitol crystals (B). There is no TTCP present in A. The peak marked with an asterisk (A) is residual agate powder from grinding TTCP and DCPA in the agate jar with agate balls for 24 h.

The present study used a finer TTCP, i.e., 5 μm median size, than the TTCP used in several previous studies [12, 20, 24]. This may explain why there were no residual TTCP present in the 24 h set CPC sample (Fig. 3), in contrast to CPC materials prepared using larger (17 μm) TTCP particles. The finer TTCP required the use of a lower P/L ratio of 2.5 compared to P/L of 3.5 to 4.0 in the previous studies. This led to a higher microporosity (47 vol %) compared to that (34 vol %) of the product prepared at P/L = 4 [24].

Incorporating 10 mass % of mannitol into the CPC powder produced a macroporosity (MacP) of 15.6 vol %, which led to an increase in dissolution rate of about 1.5 times, in terms of the rate of mass lost. A further increase in mannitol content, from 10 mass % to 50 mass %, produced a 3.5-fold increase in MacP, from 15.6 vol % to 54.7 vol %. This, however, led to a mere 1.2-fold further increase in dissolution rate, or about 1.8 times that of the non-macroprous CPC. This may be because the surface area produced by the macropores does not increase linearly with the MacP. When the dissolution rates are expressed in terms of the rate of mass loss, the CPC materials with (10 and 50) mass % mannitol dissolved at rates that were about 1.7 and 3.9 times faster than the non-macroporous CPC. Since implant resorption *in vivo* is characterized by volume changes, the results suggest that the two macroporous CPC materials should resorb close to 2 to 4 times faster than pure CPC.

Previous studies [19, 24] showed that CPC resorption rates increased significantly with decreasing Ca/P ratio of the material. This and the present finding that resorption rate increased significantly with MacP should allow formulation of CPC materials with a very wide range of *in vivo* resorption rates. Since both the lower Ca/P ratio and high porosity significantly decrease the mechanical properties of CPC but at different rates, both factors should be considered when designing CPC implants with a desired resorption rate while minimizing the loss in strengths.

## 5. Conclusions

Total porosity and macroporosity of CPC increased with increasing amount of added mannitol crystals. The dissolution rate of CPC increased with increasing amount of mannitol crystals. Increasing macropores and dissolution rates in CPC by adding mannitol crystals may provide space to facilitate bone growth.

## Figures and Tables

**Table 1:** Density and porosities of CPC specimens prepared from cement powders containing different amounts of mannitol (mean ± standard deviation; n = 5 for density, n = 3)

Group (Mannitol added)	Density (g/cm^3^)	MacP (vol %)	MicP (vol %)	Total porosity (vol %)
0 mass%	1.68 ± 0.03	0	46.8 ± 0.8	46.8 ± 0.8
10 mass%	1.41 ± 0.06	15.6 ± 3.9	39.4 ± 1.8	55.0 ± 2.1
50 mass%	0.76 ± 0.01	54.7 ± 0.8	21.1 ± 0.4	75.8 ± 0.4

**Table 2:** Mean dissolution rates of various samples prepared from cement powders containing different amounts of mannitol (mean ± standard deviation; n = 5)

Group (Mannitol added)	Rate of Mass Loss (μg/cm^2^ ×min)	Rate of Volume Loss (μL/cm^2^ ×min)
0 mass%	30.6 ± 3.4	0.018 ± 0.002
10 mass%	44.8 ± 10.2	0.032 ± 0.007
50 mass%	54.7 ± 3.6	0.072 ± 0.005

## Footnotes

1 Certain commercial materials and equipment are identified in this paper to specify the experimental procedure. In no instance does such identification imply recommendation or endorsement by the National Institutes of Health or American Dental Association Foundation or National Institute of Standards and Technology or Nihon University or that the material or equipment identified is necessarily the best available for the purpose.
